# Identification of possible targets of the *Aspergillus fumigatus CRZ1 *homologue, CrzA

**DOI:** 10.1186/1471-2180-10-12

**Published:** 2010-01-15

**Authors:** Frederico M Soriani, Iran Malavazi, Marcela Savoldi, Eduardo Espeso, Taísa M Dinamarco, Luciano AS Bernardes, Márcia ES Ferreira, Maria Helena S Goldman, Gustavo H Goldman

**Affiliations:** 1 Centro de Ciência e Tecnologia do Bioetanol and Faculdade de Ciências Farmacêuticas de Ribeirão Preto Universidade de São Paulo, São Paulo, Ribeirão Preto, 14040-903, Brazil; 2 Faculdade de Filosofia, Ciências e Letras de Ribeirão Preto, Universidade de São Paulo, São Paulo, Ribeirão Preto, 14040-903, Brazil; 3 Departamento de Genética e Evolução, Centro de Ciências Biológicas e da Saúde (CCBS), Universidade Federal de São Carlos, São Paulo, São Carlos, 13565-905, Brazil; 4 Departamento de Microbiología Molecular, Centro de Investigaciones Biológicas CSIC, Ramiro de Maeztu, 9. Madrid 28040, Spain

## Abstract

**Background:**

Calcineurin, a serine/threonine-specific protein phosphatase, plays an important role in the control of cell morphology and virulence in fungi. Calcineurin regulates localization and activity of a transcription factor called CRZ1. Recently, we characterize *Aspergillus fumigatus CRZ1 *homologue, AfCrzA. Here, we investigate which pathways are influenced by *A. fumigatus *AfCrzA during a short pulse of calcium by comparatively determining the transcriptional profile of *A. fumigatus *wild type and *ΔAfcrzA *mutant strains.

**Results:**

We were able to observe 3,622 genes modulated in at least one timepoint in the mutant when compared to the wild type strain (3,211 and 411 at 10 and 30 minutes, respectively). Decreased mRNA abundance in the *ΔcrzA *was seen for genes encoding calcium transporters, transcription factors and genes that could be directly or indirectly involved in calcium metabolism. Increased mRNA accumulation was observed for some genes encoding proteins involved in stress response. AfCrzA overexpression in *A. fumigatus *increases the expression of several of these genes. The deleted strain of one of these genes, AfRcnA, belonging to a class of endogenous calcineurin regulators, calcipressins, had more calcineurin activity after exposure to calcium and was less sensitive to menadione 30 μM, hydrogen peroxide 2.5 mM, EGTA 25 mM, and MnCl_2 _25 mM. We constructed deletion, overexpression, and GFP fusion protein for the closely related *A. nidulans *AnRcnA. GFP::RcnA was mostly detected along the germling, did not accumulate in the nuclei and its location is not affected by the cellular response to calcium chloride.

**Conclusion:**

We have performed a transcriptional profiling analysis of the *A. fumigatus ΔAfcrzA *mutant strain exposed to calcium stress. This provided an excellent opportunity to identify genes and pathways that are under the influence of AfCrzA. AfRcnA, one of these selected genes, encodes a modulator of calcineurin activity. Concomitantly with *A. fumigatus AfrcnA *molecular analysis, we decided to exploit the conserved features of *A. nidulans *calcineurin system and investigated the *A. nidulans *AnRcnA homologue. *A. nidulans *AnRcnA mutation is suppressing CnaA mutation and it is responsible for modulating the calcineurin activity and mRNA accumulation of genes encoding calcium transporters.

## Background

The phosphatase calcineurin is a heterodimeric protein composed by a catalytic subunit A and a regulatory subunit B [1]. In fungi, calcineurin plays an important role in the control of cell morphology and virulence [1-4]. Calcineurin regulates morphogenesis, Ca^+2 ^homeostasis, and stress-activated transcription in *Saccharomyces cerevisiae *[1,5]. In pathogenic fungi, calcineurin affects virulence, morphogenesis, and antifungal drug action [1,6-9]. Inactivation of calcineurin in *Cryptococcus neoformans *affects growth at 37°C and hyphal elongation during mating and haploid fruiting [10-13]. Reduced virulence and absence of growth in serum are also observed in *Candida albicans *depleted in the calcineurin activity [11,14,15]. In *A. fumigatus*, calcineurin inactivation decreases the virulence and provides decreased filamentation and no growth in serum [9,16].

Calcineurin regulates the localization and activity of the transcription factor Crz1p by dephosphorylating it [17]. Upon increase in cytosolic calcium, calcineurin dephosphorylates Crz1p, allowing its nuclear translocation [17,18]. Crz1p has a C2H2 zinc finger motif that binds to a CDRE (calcineurin-dependent response element) in the promoters of genes that are regulated by calcineurin and calcium [19]. Mutants of *S. cerevisiae *inactivated in *CRZ1 *display hypersensitivity to chloride and chitosan, a defective transcriptional response to alkaline stress, and cellular morphology and mating defects [17,19-21]. Inactivation *CRZ1 *mutants of *Schizosaccharomyces pombe *(*Δprz1*) are hypersensitive to calcium and have decreased transcription of the Pmc1 Ca^+2 ^pump [22]. *C. albicans *homozygotes *crz1Δ/Δ *are moderately attenuated for virulence and sensitive to calcium, lithium, manganese, and sodium dodecyl sulfate [18,23,24]. *A. fumigatus CRZ1 *mutant, *ΔcrzA*, is avirulent and has decreased conidiation [16,25]. Its hypersensitivity to calcium and manganese is probably due to the reduced expression of calcium transporter mRNAs under high concentrations of calcium [16].

Here, we investigate which pathways are influenced by *A. fumigatus *AfCrzA during a short pulse of calcium by comparatively determining the transcriptional profile of *A. fumigatus *wild type and *ΔAfcrzA *mutant strains. Our results revealed several possible novel targets for AfCrzA, including AfRcnA, a member of the conserved calcineurin-binding proteins, the calcipressins. Besides the transcriptional profiling of the *A. fumigatus ΔAfcrzA*, we also showed the molecular characterization of *Aspergilli *RcnAs.

## Results and Discussion

### Transcriptional profiling of the *A. fumigatus ΔcrzA *mutant strain

To have a better understanding of which genes are influenced by *A. fumigatus *AfCrzA during exposure to calcium, we performed competitive microarray hybridizations using RNA obtained from the wild type and *ΔAfcrzA *strains after short pulses (10 and 30 minutes) of 200 mM calcium chloride. RNA obtained from wild type mycelia exposed to 10 and 30 minutes calcium was taken as reference. Thus, total RNA extracted from these cultures was used to synthesize fluorescent-labeled cDNAs for competitive microarray hybridizations. In these experiments, the main aim was to focus on genes that have increased or decreased mRNA expression in the absence of AfCrzA. We were able to observe 3,622 genes modulated in at least one timepoint in the mutant when compared to the wild type strain (3,211 and 411 at 10 and 30 minutes, respectively). The large difference between the number of genes modulated at 10 and 30 minutes (about eight-times more at 10 minutes) suggests *A. fumigatus *responds rapidly to calcium stressing conditions. The full dataset was deposited in the Gene Expression Omnibus (GEO) from the National Center of Biotechnology Information (NCBI) with the number GSE15432 http://www.ncbi.nlm.nih.gov/projects/geo/query/acc.cgi?acc=GSE15432. Previously, to evaluate the effect of calcium on global *A. fumigatus *gene expression, we performed competitive microarray hybridizations using RNA obtained from the wild type strain before and after a short pulse (10 minutes) of 200 mM calcium chloride (Soriani *et al*., 2008). Statistical analysis of this dataset identified a total of 863 genes that displayed modulation. The large difference in genes whose expression is modulated between this dataset and the current dataset using a comparison between *ΔcrzA *and the wild type strains emphasizes the pleiotropic role played by *A. fumigatus *calcineurin-CrzA in the calcium-mediated signal transduction pathways. We are currently investigating the importance of this difference.

We observed differential regulation of genes involved in a variety of cellular processes and specific modulation of these functions is therefore likely to be implicated with *A. fumigatus *adaptation to high concentrations of calcium (Additional files 1 and 2, Tables S1 and S2, respectively, show the genes with log ratios more than or equal to 1 or less than or equal to 1 in at least in one time point, respectively). These genes were classified into COG functional categories http://www.ncbi.nlm.nih.gov/COG/. However, we were not able to observe any significant enrichment for a specific COG category (Additional files 1 and 2, Tables S1 and S2, repectively). We noted decreased mRNA abundance in the *ΔAfcrzA *of several genes involved in calcium transport, such as the vacuolar H^+^/Ca^+2 ^(Afu2g07630), calcium-translocating P-type ATPase (PMCA-type, Afu3g10690), and calcium-transporting ATPase 1 (PMC1, Afu7g01030). We also observed decreased mRNA accumulation when the *ΔAfcrzA *strain was exposed to calcium of genes encoding several transcription factors [CtfA (Afu4g03960), RfeF (Afu4g10200), and ZfpA (Afu8g05010)], and genes that could be directly or indirectly involved in calcium metabolism [such as a phospholipase D (Afu2g16520), two peptidyl-prolyl cis-trans isomerases (Afu5g13350 and Afu2g03720), a calcineurin binding protein (Afu2g13060), a Bar adaptor protein (Afu3g14230), and a potential regulator of cytoskeleton and endocytosis, homologue of mammalian amphiphysin. Interestingly, a chitin synthase A (Afu2g01870) also showed decreased mRNA accumulation in the *ΔAfcrzA *strain background. Cramer *et al*. [26] have shown that the calcineurin pathway plays an important role in cell wall biosynthesis in *A. fumigatus*, and that calcineurin and AfCrzA inactivation mutants are more sensitive to specific cell wall inhibitors, such as caspofungin. However, in contrast to our results these authors have observed an increased and decreased mRNA accumulation of chitin synthase A in the *ΔAfcrzA *and *ΔAfcalA *mutant strains, respectively. Several of the genes above mentioned (such as Afu2g16520, Afu2g13060, Afu3g14230, Afu4g10200, Afu8g05010, and Afu3g10690) have also been observed by Soriani *et al*. [16] as more expressed upon exposure of *A. fumigatus *to calcium. We have also previously observed that *zfpA *(Afu8g01050) has increased mRNA accumulation that is dependent on the cyclic AMP-protein kinase A signaling pathway during adaptation to voriconazole [26]. Thus, it is plausible that *zfpA *is related to a transcriptional network controlled by calcineurin-CrzA that has a key role in mediating cellular stress responses.

We observed increased mRNA accumulation when the *ΔAfcrzA *strain was exposed to calcium of genes encoding a class V chitinase (Afu7g08490), an exo-β-1,3-glucanase (Afu2g00430), an AAA family ATPase (Afu4g04800), a cation diffusion facilitator 3, a multidrug resistance protein (Afu4g01140), a TOR signalling pathway protein TipA (Afu2g07540), an inositol polyphosphate phosphatase (Afu5g02140), a representative of the Hsp9-12 heat shock protein Scf1 (Afu1g17370), and a protein phosphatase 2C (Afu4g00720). Again, the increased expression of the chitinase and exo-β-1,3-glucanase could help to explain the increased sensitivity of *A. fumigatus ΔAfcrzA *strain to caspofungin [26]. Interestingly, some of these genes are involved in stress response, such as: (i) the Scf1 homologue (Afu117370), a plasma membrane localized protein that protects membranes from desiccation and it is induced by heat shock, oxidative stress, osmostress, stationary phase entry, glucose depletion, oleate and alcohol, and is regulated by the HOG and Ras-Pka pathways http://www.yeastgenome.org[27]; (ii) TipA (Afu2g07540), a component of the TOR (target of rapamycin) signaling pathway, that interacts physically and genetically with Tap42p, which regulates protein phosphatase 2A [28]; and (iii) a protein phosphatase 2C (Afu4g00720), important physiological regulator of cell growth and of cellular stress signaling [29]. The increased mRNA accumulation of these genes could mean that they are directly or indirectly repressed by AfCrzA and can open new frontiers for studying biochemical pathways that are under influence of the *A. fumigatus *calcineurin-CrzA pathway.

**Figure 1:**
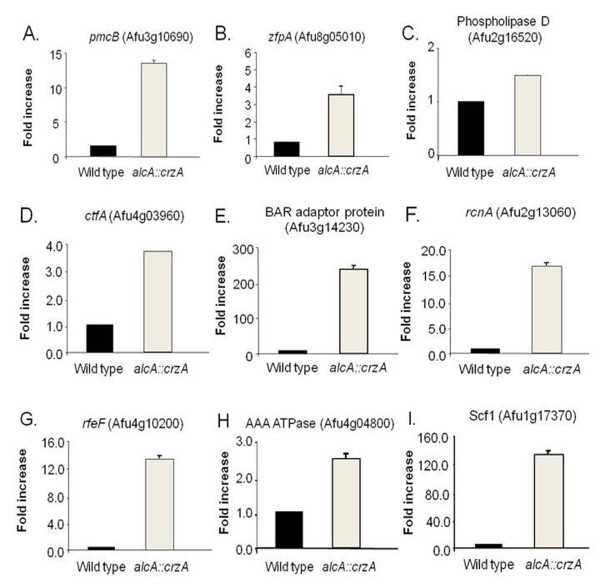
**Validation of the genes observed as more or less induced in the *A. fumigatus ΔAfcrzA *mutant**. Fold increase in mRNA levels after the incubation with 200 mM CaCl_2 _for 10 and 30 minutes of (A) *AfrcnA *(Afu2g13060), (B) *AfrfeF *(Afu4g10200), (C) Af BAR adaptor protein (Afu3g14230), (D) Af AAA ATPase (Afu4g04800), and (E) AfScf1 (Afu1g17370). Real-time RT-PCR was the method used to quantify the mRNA. The measured quantity of the mRNA in each of the treated samples was normalized using the C_T _values obtained for the β-tubulin (Afu1g10910) mRNA amplifications run in the same plate. The relative quantitation of all the genes and tubulin gene expression was determined by a standard curve (i.e., C_T _-values plotted against logarithm of the DNA copy number). The results are the means ± standard deviation of four sets of experiments. The values represent the number of times the genes are expressed compared to the corresponding control strain grown before adding 200 mM CaCl_2 _(represented absolutely as 1.00).

We then used RT-PCR, both to assess the reliability of the microarray hybridizations and to document mRNA expression from selected genes of interest in the wild type, *ΔAfcalA *and *ΔAfcrzA *mutant backgrounds, choosing five genes (three and two with decreased and increased mRNA accumulation in the microarray experiments, respectively) to analyze after 10 and 30 minutes in the presence of 200 mM CaCl2. We had previously shown that five other genes (such as Afu8g05010, C2H2 finger domain protein; Afu4g03960, C6 transcription factor; Afu2g16520, phospholipase D; and Afu2g05330, vacuolar H^+^/Ca^+2 ^exchanger; and Afu2g13060, calcineurin binding protein), identified here in this screening had induced mRNA accumulation in the presence of calcium, but reduced in *ΔAfcrzA *mutant background [16]. Expressing the results as fold induction relative to expression prior to calcium exposure, all values were in strong agreement with array hybridisation data (Figure 1). Comparisons between the gene expression variation estimated by microarray and real-time RT-PCR were performed by calculating both Pearson's (*R*_P_) and Spearman's (*R*_S_) correlation coefficients for the log2 ratios obtained by these two approaches. Positive correlation was observed for both *R*_P _and *R*_S _in all nine genes (Figure 1) [16]. Furthermore, the value of either *R*_P _or *R*_S _was above 0.70 (indicating moderate-to-strong correlation). Thus, these data suggest that our approach was able to provide information about *A. fumigatus *gene expression modulation with a considerably high level of confidence.

It is very impressive the mRNA accumulation levels of the Hsp9-12 heat shock protein Scf1 homologue (Afu1g17370): about 100 and 1000 times more in the *ΔcrzA *and *ΔcalA *than in the wild type, respectively (Figure 1E). *A. fumigatus *has two Hsp12 homologues, Afu1g17370 (e-value = 3.7e-10; 45 and 57 identity and similarity, respectively) and Afu6g12450 (e-value = 3.1e-9; 39 and 56 identity and similarity, respectively). Interestingly, the *S. cerevisiae HSP12 *was also shown to be induced by calcium but in contrast to the *A. fumigatus *homologue, the *S. cerevisiae *gene is repressed when calcium+FK506 were added and accordingly repressed in the *ΔCRZ1 *background [30]. Thus, it remains to be determined the roles played by calcineurin, AfCrzA, and AfHsp12p during adaptation of *A. fumigatus *to calcium stress.

Recently, Hagiwara *et al*. [31] identified and characterized the *A. nidulans *An*crzA *gene. They performed an *in silico *analysis by using MEME (Motif-based sequence analysis tools; http://meme.sdsc.edu/meme4_1_1/intro.html) of the possible presence of a CDRE-like consensus motif in the promoter regions of 25 AnCrzA-dependent genes. By analyzing their promoter regions, 5'-G[T/G]GGC[T/A]G[T/G]G-3' was presumed to be the consensus sequence for the *A. nidulans *AnCrzA-dependent genes. By using a combination of MEME analysis and the *A. nidulans *CDRE consensus as a guide, we were able to identify in the *AfrcnA*, *AfrfeF*, AfBAR, and the *A. fumigatus *phospholipase D promoter regions (about 500 bp upstream ATG) the following CDRE motifs: (i) *AfrcnA *(5'-GTTGGTGAG-3', -314 bp upstream ATG starting point), (ii) *AfrfeF *(5'GTGGCTGAT-3', -184 bp upstream ATG), (iii) AfBAR (5'-GTGGCTGAC-3', -309 bp upstream ATG), and (iv) *A. fumigatus *phospholipase D (5'-GTTGGAGAG-3', -239 upstream ATG). We compared these motifs with the promoter regions (about 500 bp upstream ATG) of 32 repressed genes described in Additional file 1, Table S1, and this analysis suggested 5'-GT[T/G]G[G/C][T/A]GA[G/T]-3' as the CDRE-consensus sequence for *A. fumigatus *AfCrzA-dependent genes. We also analyzed *Afscf1 *and Af AAA ATPase genes and found the following CDRE-like motifs: (i) *Afscf1 *(5'-GGGAACGAA-3', -376 bp upstream ATG), and (ii) Af AAA ATPase (5'-GAAGACGAG-3', -19 bp upstream ATG). Again, we compared these motifs with the promoter regions (about 500 bp upstream ATG) of 109 induced genes described in Additional file 2, Table S2, and we were able to propose as a putative CDRE consensus for gene repressed by *AfCrzA*, the sequence 5'-G[A/G][A/G][A/G]ACGA[A/G]-3'. It remains to be experimentally determined if these sequences are really important for AfCrzA gene regulation, both for gene induction and repression.

Prior to this work, a study analysing global gene expression regulated by the calcineurin/Crz1p signaling pathway in *S. cerevisiae *had attempted to identify genes regulated by calcium and sodium [30]. Calcineurin activation induced 153 genes involved in cell wall biosynthesis, ion homeostasis, vesicle trafficking, lipid synthesis, and protein degradation. A notable similarity was observed by the authors in the gene expression patterns of FK506-treated cells and *crz1 *cells, suggesting that Crz1p is required for most calcineurin-dependent changes in gene expression. Recently, Soriani *et al*. [16] opted to an alternative strategy, exposing *A. fumigatus *wild type strain to a short pulse with a high concentration of calcium, and arbitrarily choosing several genes that were less or more expressed in the microarray hybridization analyses to verify their expression in the wild type, and *ΔAfcalA *and *ΔAfcrzA *mutant strains by real-time RT-PCR. Thus, these authors were able to determine if the expression of these genes was dependent on calcineurin and/or AfCrzA. They verified that the majority of these genes suffered blocking of mRNA accumulation in the *ΔAfcrzA *background. The results shown here added more information about the transcriptional network involved in the calcineurin-AfCrzA in response to calcium.

### Construction of *Aspergilli *CrzA overexpression strains

**Figure 2:**
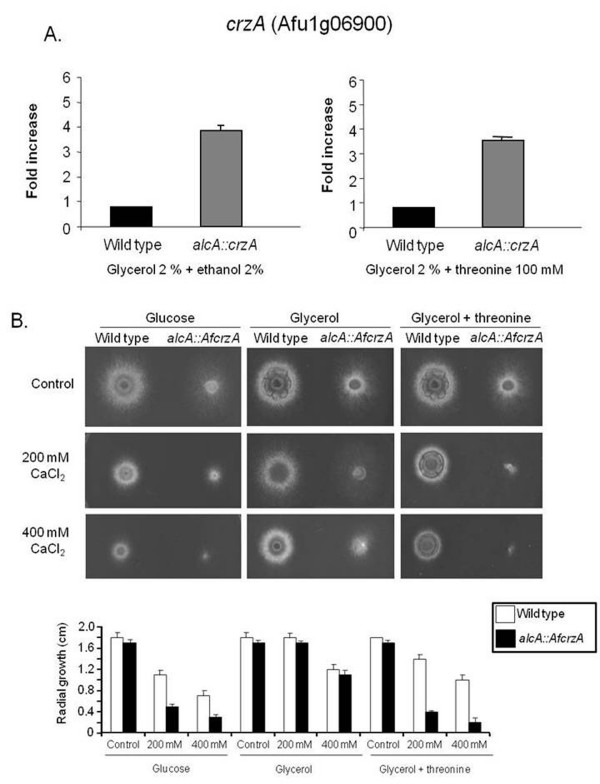
**Growth phenotypes of *A. fumigatus alcA::AfcrzA *strains grown in the presence of different calcium concentrations**. (A) Fold increase in *AfcrzA *mRNA levels after the growth of the wild type and *alcA::AfcrzA *strain either in MM+glycerol 2%+ethanol 2% or MM+glycerol 2%+threonine 100 mM for 6 hours at 37°C. The relative quantitation of *AfcrzA *and tubulin gene expression was determined by a standard curve (*i.e*., C_T_-values plotted against logarithm of the DNA copy number). The results are the means ± standard deviation of four sets of experiments. The values represent the number of times the genes are expressed compared to the corresponding wild type control strain (represented absolutely as 1.00). (B) *A. fumigatus *wild type and *alcA::AfcrzA *strains were grown for 48 hours at 37°C in MM+ 4% glucose, MM+2% glycerol, MM+2% glycerol+100 mM threonine in the absence of presence of calcium chloride 200 mM and 400 mM. The graph shows the radial growth (cm) of the strains under different growth conditions. The results are the means ± standard deviation of four sets of experiments.

**Figure 3:**
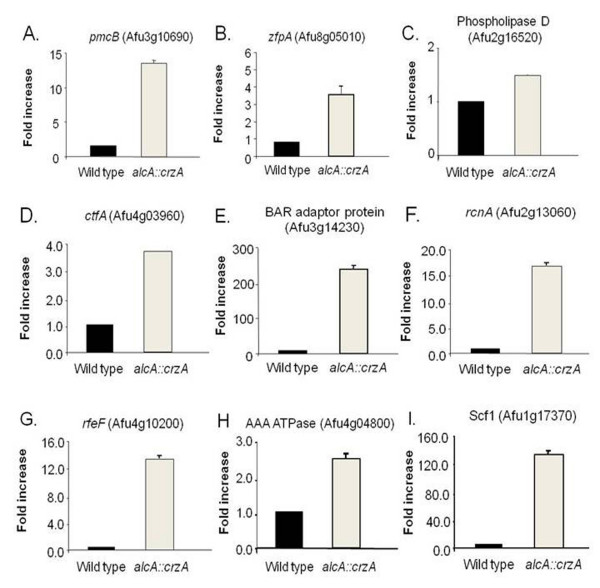
**Overepression of *A. fumigatus *AfCrzA increased the mRNA accumulation of several genes**. Fold increase in mRNA levels after the growth of the wild type and *alcA::crzA *mutant strain in MM+2% glycerol+2% ethanol for 6 hours at 37°C of (A) *AfpmcB *(Afu3g10690), (B) *AfzfpA *(Afu8g05010), (C) *A. fumigatus *Phospholipase D (Afu2g16520), (D) *AfctfA *(Afu4g03960), (E) Af BAR adaptor protein (Afu3g14230), (F) *AfrcnA *(Afu2g13060), (G) *AfrfeF *(Afu4g10200), (H) Af AAA ATPase (Afu4g04800), and (I) *Afscf1 *(Afu1g17370). The relative quantitation of all the genes and tubulin gene expression was determined by a standard curve (i.e., C_T _-values plotted against logarithm of the DNA copy number). The results are the means ± standard deviation of four sets of experiments. The values represent the number of times the genes are expressed compared to the corresponding wild type control strain (represented absolutely as 1.00).

Overexpression of AfCrzA could reveal genes regulated by the calcineurin-AfCrzA pathway. Accordingly, we constructed an overexpression *A. fumigatus *AfCrzA strain by using the *alcA *promoter. The *A. nidulans alcA *promoter homologously replaced the *AfcrzA *promoter (for details of the construction, see Methods section). The *alcA *promoter is repressed by glucose, derepressed by glycerol and induced to high levels by ethanol or L-threonine [32]. When *A. fumigatus *is grown on glycerol 2% supplemented either with ethanol 2% or threonine 100 mM, *AfcrzA *mRNA accumulation in increased about 3.8- and 3.6-times, respectively compared to growth on glucose 4% (Figure 2A, left and right graphs). As expected, when the *AfcrzA *is repressed in the presence of glucose, the *alcA::AfcrzA *strain is more sensitive to calcium (Figure 2B); however, surprisingly high levels of AfCrzA mRNA accumulation also make the *alcA::AfcrzA *strain more calcium-sensitive (Figure 2B). These results suggest that CrzA overexpression could potentially disturb the mRNA accumulation of genes that are important for the calcium homeostasis in the cell, thus disturbing the calcium metabolism into the cell and consequently the growth in the presence of increasing calcium concentrations. To test this hypothesis we used real-time RT-PCR to verify the mRNA levels of selected genes observed as having decreased mRNA and increased accumulation (see Additional file 1, Table S1). As it can be seen in Figure 3, all tested genes to different extent have increased mRNA accumulation when the *AfcrzA *is overexpressed. The Af AAA ATPase (Afu4g04800) and AfScf1 (Afu1g17370) have increased mRNA accumulation when the *ΔAfcrzA *was exposed to calcium suggesting they are repressed by AfCrzA (see Figures 1E-F). We expected their mRNA accumulation would be reduced when AfCrzA is overexpressed. However, the mRNA levels of these genes were lower in the *alcA::AfcrzA *than in the *ΔAfcrzA *mutant strain (compare Figures 1E-F with Figures 3I-J), what could indicate that AfCrzA is partially controlling the mRNA accumulation levels of these genes. The genes encoding *AfpmcB *(Afu3g10690), *AfrcnA *(Afu2g13060), *AfrfeF *(Afu4g10200), and AfBAR adaptor protein (Afu3g14230) are about 14, 16, 13, and 250 times more expressed in the *alcA::AfcrzA *mutant than the wild type strain, respectively (Figure 3B and 3F-H). The *AfpmcB *(Afu3g10690) gene is *A. fumigatus *homologue of the yeast *PMC1*, a vacuolar Ca^+2 ^ATPase involved in depleting cytosol of Ca^+2 ^ions and preventing growth inhibition by activation of calcineurin in the presence of elevated concentrations of calcium [33]. The increased expression of this gene suggests AfCrzA is controlling directly or indirectly its expression. Furthermore, the increased mRNA accumulation of these calcium transporter-encoding genes is quite consistent taking into consideration the dramatic stress condition caused by the sudden increase in calcium concentration that needs either to be removed from the cytoplasm or transported to vacuoles. Considering the growth inhibition of the *Aspergilli alcA::AfcrzA *strain under high-Ca^+2 ^conditions (see Figures 2A-B), one possible interpretation of these results is that the AfCrzA overexpression can inhibit the function of another factor that is necessary for growth under high-Ca^+2 ^conditions.

*A. fumigatus *AfRcnA belongs to a class of endogenous calcineurin regulators, calcipressins, a family of calcineurin-binding proteins, conserved from yeast to mammals [34,35]. A phylogenetic analysis was performed to determine the relationship of AfRcnA to calcipressin homologues in several different organisms (Additional file 3, Figure S1). The mechanism how this protein family functions still remains controversial. There are reports showing that calcipressins can both stimulate and inhibit the calcineurin pathway 34,35,36. Induction of *S. cerevisiae RCN1-lacZ *in response to calcium was completely blocked by addition of FK506 or by deletion of the genes encoding Tcn1p or calcineurin [33]. The *S. cerevisiae RCN1 *is also induced by calcium, repressed by calcium+FK506 and in the *crz1 *background [30]. Another member of this family, Cbp1, was identified in *Cryptococcus neoformans*, and is required for mating but not for growth at 37°C [37]. We have observed that *AfrcnA *mRNA accumulation upon calcium stress is dependent on both calcineurin and AfCrzA (Figure 1A). These results suggest that both *S. cerevisiae *and *A. fumigatus RCAN *homologues may be downstream targets of the calcineurin-dependent transcription factor. This fits a model where increased *A. fumigatus *AfRcnA regulation in response to calcineurin signaling is possibly a negative-feedback mechanism modulating calcineurin acitivity.

We constructed an *A. nidulans alcA::AncrzA *also by replacing the endogenous *AncrzA *promoter region homologously with the *A. nidulans alcA *promoter. We investigate the genetic interactions between *ΔAncnaA *and *ΔAncrzA *mutations and a double mutant *ΔAncnaA ΔAncrzA *displays the same growth behavior than the *ΔAncnaA *mutant indicating as expected that *AncnaA *is epistatic to *AncrzA *(data not shown).

### Molecular characterization of the *Aspergilli *calcipressin homologue

**Figure 4:**
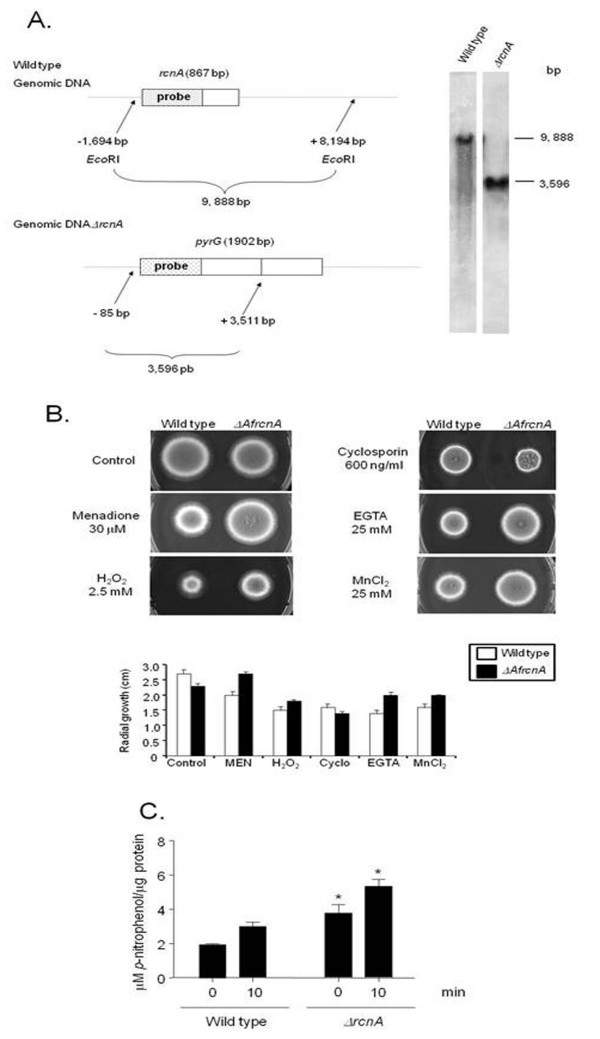
**Molecular characterization of the *A. fumigatus AfrcnA***. (A) Schematic illustration of the *rcnA *deletion strategy. (A) Genomic DNA from both wild type and *ΔAfrcnA *strains was isolated and cleaved with the enzyme *Eco*RI; a 2.0-kb DNA fragment from the 5'-noncoding region was used as a hybridization probe. This fragment recognizes a single DNA band (about 9.8-kb) in the wild type strain and also a single DNA band (about 3.6-kb) in the *ΔrcnA *mutant as shown in the Southern blot analysis. (B) Wild type and *ΔAfrcnA *mutant strains were grown for 72 hours at 37°C in complete medium in the absence or presence of menadione 30 μM, H_2_O_2 _2.5 mM, cyclosporine A 600 ng/ml, EGTA 25 mM, and MnCl_2 _25 mM. The graph shows the radial growth (cm) of the strains under different growth conditions. The results are the means ± standard deviation of four sets of experiments. (C) Wild type and *ΔrcnA *mutant strains were grown in YG medium for 16 hours at 37°C and then exposed to 200 mM CaCl_2 _for 10 minutes. Mycelial protein extracts were processed and calcineurin activity measured. Asterisks indicate the *ΔrcnA *samples are significantly different from the wild type strain (p < 0.05).

To have more information about the function of some of the genes identified as more expressed in the *A. fumigatus *wild type and repressed in the *ΔAfcrzA*, we inactivated the *AfrcnA *(Afu2g13060), *AfrfeF *(Afu4g10200), Af BAR adaptor protein (Afu3g14230), and *A. fumigatus *phospholipase D (Afu2g16520). Since calcium is involved in different kinds of stresses, such as oxidative stress and uncontrolled proliferation and survival [38-44], we decided to determine if several different culture conditions could affect the growth of these deletion strains. Except for *ΔAfrcnA*, the deletion mutants showed comparable growth phenotypes to the wild type strain in the presence of the following agents or stressing situations: oxidizing agents and metals (paraquat, *t*-butyl hydroperoxide, zinc, iron, and chromium), calcium, cyclosporine A, DNA damaging agents (4-nitroquinoline oxide, hydroxyurea, camptothecin, and bleomycin), and temperature (30, 37, and 44°C) (data not shown). However, *ΔAfrcnA *growth was less sensitive to menadione 30 μM, hydrogen peroxide 2.5 mM, EGTA 25 mM, and MnCl_2 _25 mM (Figure 4B). We exposed both wild type and *ΔAfrcnA *strains for 200 mM calcium chloride for 10 minutes and measured the calcineurin activity in these strains (Figure 4C). In the wild type strain, there is about 50% increase in the calcineurin activity when the mycelia was exposed to calcium chloride 200 mM for 10 minutes (Figure 4C). However, in the *ΔAfrcnA *mutant strain there is a significant increase in the calcineurin activity at 0 and 10 minutes in the presence of calcium chloride (Figure 4C). These results suggest that AfRcnA has an inhibitory effect on calcineurin activity when *A. fumigatus *is exposed to high calcium concentrations.

**Figure 5:**
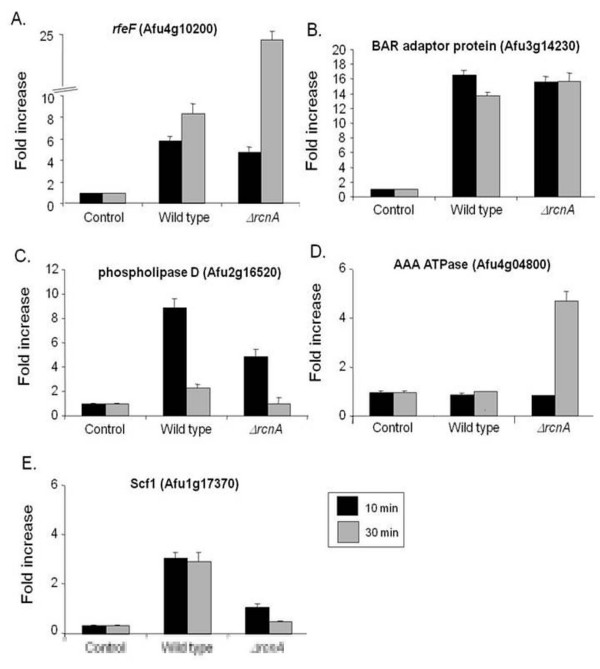
**AfRcnA affects the mRNA accumulation of genes whose expression is influenced by *AfcrzA***. Fold increase in mRNA levels after the incubation ot he wild type and *ΔAfrcnA *mutant strains with 200 mM CaCl_2 _for 10 and 30 minutes of (A) *AfrfeF *(Afu4g10200), (B) Af Bar adaptor protein (Afu3g14230), (C) *A. fumigatus *phospholipase D (Afu2g16520), (D) Af AAA ATPase (Afu4g04800), and (E) *Afscf1 *(Afu1g17370). The relative quantitation of all the genes and tubulin gene expression was determined by a standard curve (i.e., C_T _-values plotted against logarithm of the DNA copy number). The results are the means ± standard deviation of four sets of experiments. The values represent the number of times the genes are expressed compared to the corresponding wild type control strain (represented absolutely as 1.00).

We also investigated how the *AfrcnA *deletion would affect the mRNA accumulation of the genes observed as modulated by AfCrzA (see Figure 1). Thus, we exposed both the wild type and *AfΔrcnA *mutant strains to CaCl_2 _200 mM for 10 and 30 minutes and measured the mRNA accumulation of these genes (Figure 5). The absence of AfRcnA has a very heterogeneous influence on the mRNA accumulation of these genes. The *AfrfeF *(Afu4g10200) and Af AAA ATPase (Afu4g04800) genes have increased mRNA accumulation in the absence of *AfrcnA *when compared to the wild type strain (about 1.5- and 5.0-times and about the same and 5-times increased at 10 and 30 minutes, respectively; Figures 5A and 5D). In contrast, the *A. fumigatus *phospholipase D (Afu2g16520) gene has lower mRNA accumulation of 3.6- and 5.0-times in the *AfΔrcnA *mutant than the wild type strain (Figure 5C). The mRNA accumulation of the Af BAR (Afu3g14230) and AfScf1 (Afu1g17370) genes is not affected by the absence of *AfrcnA *(Figures 5B and 5E). These data emphasize the complex influence of AfRcnA on the calcineurin pathway, both stimulating and inhibiting genes in this pathway.

**Figure 6:**
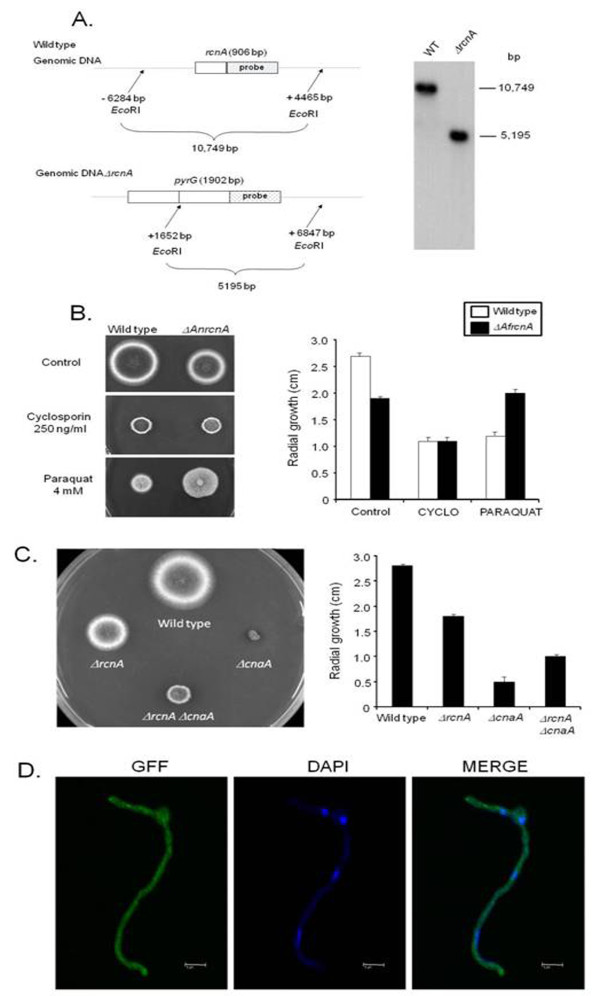
**Molecular characterization of the *A. nidulans AnrcnA *gene**. (A) Schematic illustration of the *AnrcnA *deletion strategy. (A) Genomic DNA from both wild type and *ΔAnrcnA *strains was isolated and cleaved with the enzyme *Eco*RI; a 2.0-kb DNA fragment from the 3'-noncoding region was used as a hybridization probe. This fragment recognizes a single DNA band (about 10.7-kb) in the wild type strain and also a single DNA band (about 5.2-kb) in the Δ*AnrcnA *mutant as shown in the Southern blot analysis. (B) Wild type and *ΔAnrcnA *mutant strains were grown for 72 hours at 37°C in complete medium in the absence or presence of cyclosporine A 250 ng/ml and paraquat 4 mM. (C) Growth phenotypes of *A. nidulans *wild type, *ΔAnrcnA*, *ΔAncnaA*, *ΔAncnaA *mutant strains were grown in complete medium for 72 hours at 37°C. In (B) and (C) graphs show the radial growth (cm) of the strains under different growth conditions. The results are the means ± standard deviation of four sets of experiments. (D) GFP::AnRcnA localizes to the cytoplasm. Germlings of the GFP::AnRcnA were grown in liquid MM+ 2% glycerol for 24 hs at 30°C. The germlings were treated or not with 50 mM calcium chloride for different periods of time from 5 to 60 minutes. After the treatment, germlings were analysed by laser scanning confocal microscopy. The figure shows a GFP::AnRcnA germling exposed to calcium chloride; however, germlings not exposed to calcium chloride displayed essentially the same results. Images were captured by direct acquisition. Bars, 5 μm.

After several attempts, we were unable to obtain a completely functional *A. fumigatus GFP::AfRcnA *and an overexpression *alcA:AfrcnA *strains (data not shown). Thus, we decided to exploit the conserved features of *A. nidulans *calcineurin system [see [30]] and construct both an *A. nidulans *GFP and an *alcA::AnrcnA *strain. The *A. nidulans *AnRcnA homologue (AN6249.3) has about 71% identity and 82% (e-value 3e-94) similarity with the *A. fumigatus *AnRcnA (see also Additional file 3, Figure S1). Furthermore, to have a more detailed analysis of the *A. nidulans *AnRcnA, we also constructed an *A. nidulans ΔAnrcnA *deletion strain (Figure 6A). We evaluated its phenotype by using the same strategies above outlined for the *A. fumigatus ΔAfrcnA*. The *A. nidulans ΔAnrcnA *radial diameter is about 25% smaller than the wild type strain (Figure 6B). It is also more resistant to cyclosporine A (observe both strains have the same radial diameter when grown in the presence of cyclosporine A, however *A. nidulans ΔAnrcnA *is smaller than the wild type; Figure 6B). We have observed that the deletion of *A. nidulans AnrcnA *also confers more resistance to an oxidative stressing agent, paraquat at 4 mM (Figure 6B). Interestingly, *A. nidulans *showed about 3.5 times more mRNA accumulation of the *rcnA *gene when mycelia were exposed to 0.5 mM menadione compared to mycelia not exposed to it (data not shown).

The first member identified from the calcipressin family, *RCAN1*, was isolated from the hamster genome as a gene induced during transient adaptation to oxidative stress [42,43]. It was observed that resistance to oxidative stress and calcium stress increased as a function of *RCAN1 *expression and decreased as its expression diminished [44]. Porta *et al*. [35] have shown that RCAN1 mRNA and protein expression are sensitive to oxidative stress in primary neurons, and that *Rcan1^-/- ^*neurons display an increased resistance to damage by hydrogen peroxide. Taken together, our results suggest that *Aspergilli *RcnA play a role in calcium and oxidative stress signaling.

Next step, we crossed the *A. nidulans ΔAnrcnA *strain with *ΔAncnaA *strain (*cnaA *encodes the catalytic subunit of the calcineurin gene) [30]. The *A. nidulans ΔAnrcnA *mutation can partially suppress the *ΔAncnaA *growth defect, suggesting a genetic interaction between AnRcnA and AnCnaA (Figure 6C). To determine the AnRcnA cellular localization, we transformed a GFP::AnRcnA cassette into a wild type strain. Several transformants were obtained in which the plasmid had integrated homologously at the *AnrcnA *locus (data not shown). Thus, in these strains GFP::AnRcnA is the only source of protein and GFP::AnRcnA strains are completely functional, *i.e*., they displayed cyclosporine A/paraquat-sensitivity comparable to the wild type strain (data not shown). Figure 6D shows GFP::AnRcnA germlings that were grown for 24 hs in MM+2% glycerol at 30°C and either incubated or not in the presence of calcium chloride 50 mM or EGTA 25 mM for 5 to 15 minutes. In all conditions, AnRcnA was mostly detected along the germling and did not accumulate in the nuclei (Figure 6D and data not shown). The same results were observed when glucose was used as a single carbon source (data not shown). These results show that AnRcnA cellular localization is not affected by the cellular response to calcium chloride.

**Figure 7:**
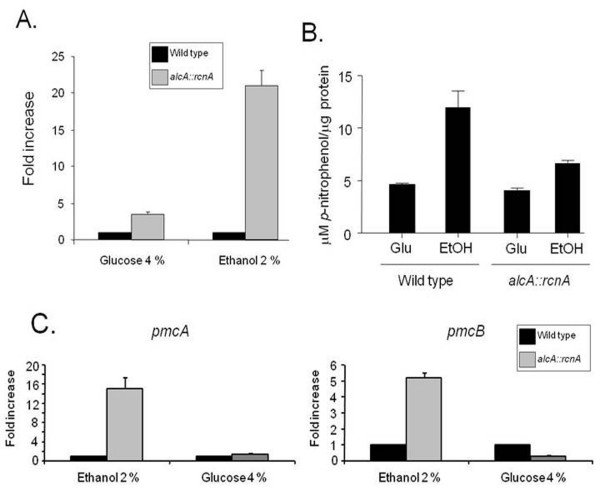
**Overexpression of the *A. nidulans AnrcnA *gene**. (A) Fold increase in *AnrcnA *mRNA levels after the growth of the wild type and *alcA::AnrcnA *mutant strain in MM+4% glucose and MM+2% glycerol+100 mM ethanol for 6 hours at 37°C. (B) Wild type and mutant strains were grown in MM+2% glycerol for 18 hours at 37°C and then transferred to either MM+4% glucose or MM+2% glycerol+2% ethanol for additional 6 hours at the same temperature. Mycelial protein extracts were processed and calcineurin activity measured. (C) A similar experiment as described in (B) was performed and *pmcA *and *pmcB *mRNA accumulation was evaluated by real-time RT-PCR. For (A) the relative quantitation of all the genes and tubulin gene expression was determined by a standard curve (i.e., C_T _-values plotted against logarithm of the DNA copy number). The results are the means standard deviation of four sets of experiments. The values represent the number of times the genes are expressed compared to the corresponding wild type control strain (represented absolutely as 1.00).

We overexpressed *AnrcnA *aiming to investigate genes that could be potentially regulated by the calcipressin-calcineurin pathway. Accordingly, we constructed an *A. nidulans *overexpression *AnrcnA *strain by using the *alcA *promoter. We used real-time RT-PCR to test the mRNA levels of *AnrcnA *when the wild type and *alcA::AnrcnA *strains were grown in the presence of either glucose or glycerol+ethanol as carbon sources (Figure 7A). The *AnrcnA *gene showed about the same mRNA accumulation when the wild type strain was grown either in the presence of glucose or glycerol+ethanol (Figures 7A). However, when the *alcA::AnrcnA *strain was grown in the presence of glycerol+ethanol, the *AnrcnA *gene had a mRNA accumulation of about 16.0 times when compared its growth in the presence of glucose (Figures 7A). Surprisingly, AnRcnA overexpression in liquid medium (for 16 hours at 37°C) did not cause any growth inhibition in the presence of either calcium or cyclosporine (data not shown). AnRcnA overexpression (for 16 hours at 37°C) also had no effect on the *ΔAncnaA *phenotype in liquid medium (data not shown). Next, we observed the effects of overexpressing AnRcnA on calcineurin activity. Interestingly, calcineurin activity is dramatically increased when the wild type strain was grown in the presence of ethanol (Figure 7B). In the *alcA::AnrcnA *strain, calcineurin activity was reduced about 50% (Figure 7B), what it is again consistent with a role for *Aspergilli *RcnAs in the inhibition of calcineurin activity. Both strains display the same calcineurin activity when grown in the presence of glucose (Figure 7B). Assuming an inhibitory role for AnRcnA on the calcineurin activity, it should be expected an increase in the calcineurin activity for the *alcA::AnrcnA *grown in the presence of glucose. However, the lack of reduction in the calcineurin activity is possibly due to the fact that the *rcnA *mRNA accumulation was not completely abolished in the *alcA::AnrcnA *grown in the presence of glucose (Figure 7A). Overexpression of the yeast protein Rcn1p or the human homologues also inhibited the activation of the transcription factor Crz1p and the inhibition of the H^+^/Ca^2+ ^exchanger Vcx1p [33].

We investigated the effects of AnRcnA overexpression on the mRNA accumulation of the calcium transporters *pmcA *(AN1189.3) and *pmcB *(AN4920.3), two *A. nidulans PMC1 *homologues. Low and about similar *pmcA *and *pmcB *mRNA accumulation were seen when the wild type and the *alcA::AnrcnA *mutant strains were grown in the presence of glucose (Figure 7C). In contrast, *pmcA *and *pmcB *levels were about 16 and 5 times higher the *alcA::AnrcnA *strain than in the wild type when both strains were grown in the presence of glycerol+ethanol (Figure 7C). These results strongly suggest that AnRcnA can directly or indirectly influence the *pmcA *and *pmcB *mRNA accumulation. Thus, it is possible RcnA has both stimulatory and inhibitory activity depending on the calcineurin pathway activation by calcium stress.

Taken together, these results strongly suggest that: (i) *rcnA *genes are involved in the oxidative stress and calcium stress in *Aspergilli*, (ii) both *AncnaA *and *AnrcnA *genes showed genetic interactions, and (iii) RcnA can modulate calcineurin activity and the mRNA accumulation of genes encoding calcium transporters. What is the nature of the interaction between *Aspergilli *CnaA and RcnA? These interactions could mean protein-protein interactions, and considering that calcipressin homologues from other species were already shown to interact with calcineurin 35,45, we investigated the possibility of AfRcnA to bind AfCnaA by using yeast two-hybrid analysis. Our results have not revealed any even weak interaction between these two proteins (data not shown), suggesting that the basis for the interaction is either not related to protein-protein interaction or alternatively there are other proteins or conditions that mediate this interactions that cannot be completely recapitulated by using yeast two-hybrid assays. The *ΔAnrcnA *mutation suppresses the *ΔAncnaA *mutation and suppression of a null allele is expected to be due to downstream mutations that activate the pathway independent of the original (suppressed) gene product [45]. This suppression is essentially visible in terms of the recovery of the colonial growth and increase in the radial diameter of the double mutant (see Figure 5C). Considering the dramatic morphological phenotype of *ΔAncnaA *strain, it is possible that besides controlling calcineurin activity, AnRcnA is also involved in *Aspergillus *development. Involvement of calcipressins in development has been previously reported for the *Drosophila melanogaster *sarah mutants [46]. Eggs laid by sarah mutant females arrest in anaphase of meiosis I and fail to fully polyadenylate and translate bicoid mRNA. Furthermore, sarah mutant eggs show elevated cyclin B levels, indicating a failure to inactivate M-phase promoting factor (MPF). Taken together, these results demonstrate that calcium signaling is involved in Drosophila egg activation. It remains to be determined the further involvement of AnRcnA in *A. nidulans *development.

During the writing of this paper, a complementary study reporting the construction of the *ΔAfrcnA *mutant in the *A. fumigatus *strain AF293 was published (named CbpA) [47]. These authors observed that deletion of the *cbpA *gene resulted in reduced hyphal growth and limited attenuated virulence. Different from our results, they also observed that the Δ*cbpA *strain showed increased calcium tolerance compared to the wild-type strain. Some differences between ours and their results can be credited to *A. fumigatus *strain differences. However, it is interesting to emphasize the fact that both *Aspergilli *showed some differences in the susceptibilities to manganese and EGTA (*A. fumigatus*) and cyclosporine A (*A. nidulans*). In contrast, those authors have shown that the *A. fumigatus *AF293 Δ*cbpA *and wild-type strains displayed an equal sensitivity to the oxidants menadione and hydrogen peroxide, and were also not able to demonstrate a direct protein-protein interaction between *A. fumigatus *CbpA and AfCnaA [47].

## Conclusion

We have performed a transcriptional profiling analysis of the *A. fumigatus ΔAfcrzA *mutant strain exposed to calcium stress. This provided an excellent opportunity to identify genes and pathways that are under the influence of AfCrzA. We validated the relationship between AfCrzA and these selected genes by using deletion analysis and by checking through real-time RT-PCR the mRNA accumulation of these genes expressed either in the *ΔAfcrzA *or overexpression strains. AfRcnA, one of these selected genes, encodes a modulator of calcineurin activity. Recently, we demonstrated that contrary to previous findings, the gene encoding the *A. nidulans *calcineurin catalytic subunit homologue, *AncnaA*, is not essential and that the *AncnaA *deletion mutant shares the morphological phenotypes observed in the corresponding *A. fumigatus *mutant, *ΔcalA *[30]. Thus, we decided once more to exploit the conserved features of *A. nidulans *calcineurin system and concomitantly with *A. fumigatus AfrcnA *molecular analysis, we investigated the *A. nidulans *AnRcnA homologue. AnRcnA showed to interact genetically with AnCnaA and be responsible for modulating the calcineurin activity and mRNA accumulation of genes encoding calcium transporters.

In summary, our work opens exciting new avenues for research into environmental sensing and nutrient acquisition mediated by the calcineurin-CrzA pathway in this important human pathogen.

## Methods

### Strains and media methods

*A. fumigatus *strains used in this study are CEA17 (*pyrG-*), CEA17-80 (wild type), Δ*calA *[9], FMS5 (Δ*crzA::pyrG*) [16], ALCCRZA (*alcA::crzA*), and RCNA (*ΔrcnA*). *A. nidulans *strains used are GR5 (*pyroA4 pyrG89; wA3*), TNO2a3 (*pyroA4 pyrG8 ΔnKUa::argB*) [49], CNA1 (*ΔcnaA::pyroA*; *pyroA4 pyrG89; wA3*) [16], ALCRZA1 (*pyroA4, alcA::gfp::crzA*), RCNA1 (*pyroA4, ΔrcnA::pyrG*), and ALCARCNA (*pyroA4, alcA::gfp::rcnA*). Media were of two basic types. A complete medium with three variants: YAG (2% glucose, 0.5% yeast extract, 2% agar, trace elements), YUU (YAG supplemented with 1.2 g/l each of uracil and uridine) and liquid YG or YG + UU medium of the same compositions (but without agar). A modified minimal medium (MM: 1% glucose, original high nitrate salts, trace elements, 2% agar, pH 6.5) was also used. Trace elements, vitamins, and nitrate salts are described by Kafer [48]. Expression of tagged genes under the control of *alcA *promoter was regulated by carbon source: repression on glucose 4% (w/v), derepression on glycerol and induction on ethanol or threonine. Therefore, MM-G and MM-E (or MM-T) were identical to MM, except that glycerol (2% v/v) and/or ethanol (2% v/v for liquid medium) or threonine (100 mM for solid medium) were used, respectively, in place of glucose as the sole carbon source. Strains were grown at 37°C unless indicated otherwise. Cyclosporine A (CsA) used in the experiments throughout the manuscript is from Neoral™ Sandimmun (Novartis). Standard genetic techniques for *A. nidulans *were used for all strain constructions [49].

### RNA isolation

For the microarray experiments, 1.0 × 10^9 ^conidia of *A. fumigatus *wild type and Δ*crzA *strains were used to inoculate 400 ml liquid cultures (YG) in 1000 ml erlenmeyer flasks that were incubated in a reciprocal shaker (250 rpm) at 37°C for 16 hours. After this period, the germlings were harvested by filtration and transferred to a fresh YG medium plus 200 mM of CaCl_2 _for either 10 or 30 minutes. Again, after this period, the germlings were harvested by centrifugation or filtration immediately frozen in liquid nitrogen. For total RNA isolation, the germlings were disrupted by grinding in liquid nitrogen with pestle and mortar and total RNA was extracted with Trizol reagent (Invitrogen, USA). Ten micrograms of RNA from each treatment were then fractionated in 2.2 M formaldehyde, 1.2% w/v agarose gel, stained with ethidium bromide, and then visualized with UV-light. The presence of intact 25S and 17S ribosomal RNA bands was used as a criterion to assess the integrity of the RNA. RNAse free DNAse I treatment for the real-time RT-PCR experiments was carried out as previously described [50].

### Assay for calcineurin activity

Cytoplasmic extracts were prepared by homogenizing the mycelia using liquid nitrogen in a buffer containing 50 mM Tris-HCl, pH7.4, 1 mM EGTA, 0.2% Triton X-100, 1 mM benzamidine, and 10 g/ml each of leupeptin, pepstatin and aprotinine. The homogenates were clarified by centrifugation at 10,000 × g for 10 min at 4°C and then at 20,800 × g for 60 min at 4°C. Protein content in the extracts was determined by the method of Bradford [51] and then used for calcineurin activity assays. Calcineurin activity in the cytoplasmic extracts was assayed according to the method of Wang and Pallen [52], with minor modifications, by determining calmodulin-dependent protein phosphatase activity in the absence or in the presence of the inhibitor CsA (5 mM). CsA is an immunosuppressant that targets calcineurin by forming a molecular complex with cytosolic protein cyclophilin of immunocompetent lymphocytes, especially T-lymphocytes. This complex of CsA and cyclophylin inhibits its phosphatase activity. Assays were performed in a reaction mixture (100- l volume) containing 25 mM Tris (pH 7.2), 25 mM MES (pH 7.0), 5 mM *p*-nitrophenyl phosphate, followed by incubation at 30°C for 10 min, and terminated by the addition of 10 l of 13% (w/v) KH2PO4. The absorbance of the samples was measured immediately at 405 nM. The difference between the amounts of *p*-nitrophenol released in the absence and the presence of ciclosporin represented the phosphatase activity mediated by calcineurin. One unit of enzyme activity is defined as nmol of *p*-nitrophenol released from *p*-nitrophenyl phosphate.min^-1^.mg protein^-1^.

### Gene Expression Methods

We have used the *A. fumigatus *oligonucleotide slides version 2 for microarray hybridizations (for details see http://pfgrc.jcvi.org/index.php/microarray/array_description/aspergillus_fumigatus/version2.html). The RNA samples extracted, as described above, were further purified with the RNA easy kit (Qiagen, Germany) and directly labelled by incorporation of Cy3- or Cy5-dUTP (GE Health Care). The resulting data was averaged from duplicate genes on each array, from dye-swap hybridizations for each experiment, and from two biological replicates, taking a total of 8 intensity data points for each gene. Differentially expressed genes at the 95% confidence level were determined using intensity-dependent Z-scores (with Z = 1.96) as implemented in MIDAS and the union of all genes identified at each time point were considered significant in this experiment. The resulting data were organized and visualized based on similar expression vectors in genes using Euclidean distance and hierarchical clustering with average linkage clustering method to view the whole data set and k-means to group the genes in 60 clusters with TIGR MEV (multi experiment viewer), also available at http://www.jcvi.org/cms/research/software. All the hybridization conditions and microarray data analysis were performed as previously described by Malavazi *et al*. [53]. When RT-PCR was used to assess the reliability of the microarray hybridizations germlings were exposed to a novel growth curve (new RNA samples, not stocks of the original RNA used in the array experiment).

### Real-time RT PCR reactions

All the PCR and RT-PCR reactions were performed using an ABI 7500 Fast Real-Time PCR System (Applied Biosystems, USA). Taq-Man™ Universal PCR Master Mix kit (Applied Biosystems, USA) was used for PCR reactions. The reactions and calculations were performed according to Semighini *et al*. [49]. The primers and Lux™ fluorescent probes (Invitrogen, USA) used in this work are described in Additional file 4, Table S3.

### Staining and microscopy

For cell imaging of RcnA fused to GFP, conidiospores were grown in glass-bottom dishes (Mattek Corporation, USA) in 2 ml of MM+2% glycerol for 24 hours at 30°C. All the confocal images were analysed using the Leica TCS SP5 laser scanning confocal microscope (Leica Microsystems, Heidelberg, Germany) (Laboratory of Confocal Microscopy, FMRP-USP, Brazil) using 63× magnification water immersion objective lens using laser lines 488 nm for GFP and 405 nm for DAPI. Images were captured by direct acquisition with the Leica LAS AF software (Leica Microsystems) and additional processing was carried out using Adobe Photoshop 7.0 (Adobe Systems Incorporated, CA).

### DNA manipulations and construction of the Aspergilli conditional mutants

DNA manipulations were according to Sambrook and Russell [54]. All PCR reactions were performed using Platinum Taq DNA Polimerase High Fidelity (Invitrogen). For the DNA-mediated transformation, the deletion cassettes were constructed by "in vivo" recombination in *S. cerevisiae *as previously described by Colot *et al*. [55]. About 2.0-kb regions on either side of the ORFs were selected for primer design. For the construction of the *A. fumigatus rcnA *deletion, the primers calp-Afu P1 and calp-Afu P2 were used to amplify the 5'-UTR flanking region of the targeted ORF. The primers calp-Afu P3 and calp-Afu P4 were used to amplify the 3'-UTR ORF flanking region. For the construction of the *A. nidulans rcnA *deletion, the primers calp-Ani P1 and calp-Ani P2 were used to amplify the 5'-UTR flanking region of the targeted ORF. The primers calp-Ani P3 and calp-Ani P4 were used to amplify the 3'-UTR ORF flanking region. Both fragments 5- and 3-UTR were PCR-amplified from genomic DNA using as templates the A4 strain for *A. nidulans *and AFU293 for *A. fumigatus *cassettes. The *pyrG *used in the *Aspergilli *cassettes for generating both deletion strains was used as marker for auxotrophy and were amplified (by using primers *pyrG *Fw and *pyrG *Rw) from pCDA21 plasmid [56].

Cassettes generation was achieved by transforming each fragment for each construction along with the plasmid pRS426 *BamHI*/*EcoRI *cut in the in *S. cerevisiae *strain SC9421 by the lithium acetate method [57]. The DNA of the yeast transformants was extracted by the method described by Goldman *et al*. [58], dialysed and transformed by electroporation in *Escherichia coli *strain DH10B to rescue the pRS426 plasmid harboring the cassettes. The cassettes were PCR-amplified from these plasmids and used for transformation of *Aspergilli *according to the procedure of Osmani *et al*. [59]. Transformants were scored for their ability to grow on minimal medium. PCR or Southern blot analyses were used throughout of the manuscript to demonstrate that the transformation cassettes had integrated homologously at the targeted *A. fumigatus *or *A. nidulans *loci.

The *A. fumigatus alcA::AfcrzA *and *A. nidulans alcA::AncrzA *constructions were performed by amplifying by PCR 5'-end fragments (for *A*. fumigatus, 1084-bp from the start codon of the ORF with the primers Afcrz1 AscI: 5'-GGCGCGCCAATGGCTTCACAGGAGATGTTCC-3' and Afcrz1 PacI: 5'-CCTTAATTAAGCACATTGGGCATCATTTCCTGTCC-3'; and for *A. nidulans*, 1068 bp from the start codon of the ORF with the primers AncrzA AscI 5'-GGCGCGCCAATGGATCCTCAAGATACGCTGCAGG-3' and AncrzA PacI 5'-CCTTAATTAACATCTGTGACGCTTGCCCGATATC-3'), digesting them with *Pac*I and *Asc*I, and cloning them in the corresponing *Pac*I and *Asc*I restriction sites of the pMCB17-apx plasmid. The fragment of the ORF is under the control of the *A. nidulans alcA *promoter and after homologous integration the translation produces an N-terminal fusion protein. *A. fumigatus *and *A. nidulans pyrG^- ^*strains were transformed with the corresponding vectors pMCB17-apx-crzA and after homologous recombination the *alcA::gfp::crzA *construction and a truncated *crzA *non-coding gene were generated. All the transformants were confirmed by PCR using specific primers.

## Authors' contributions

FMS carried out microarray hybridization experiments and analysis, construction of *A. fumigatus *deletion and overexpression strains, and real-time RT-PCR experiments. IM performed the yeast two-hybrid experiments, the construction of *alcA::rcnA *strain, the GFP microscopy, characterized the RcnA deletion and overexpression strains. MS helped and performed the real-time RT-PCR and fungal transformation experiments. LASB contributed with the bioinformatics analysis. MESF, TMD, EE and MHSG contributed to design of the experiments and discussion of the results. GHG wrote the manuscript and supervised all the work. All authors read and approved the final manuscript

## Supplementary Material

Additional file 1 **Genes less expressed after *Aspergillus fumigatus ΔcrzA *mutant CaCl_2 _200 mM exposition for 10 and 30 minutes**. List of the genes identified in the microarray experiment as less expressed. Click here for file

Additional file 2 **Genes more expressed after *Aspergillus fumigatus ΔcrzA *mutant CaCl_2 _200 mM exposition for 10 and 30 minutes**. List of the genes identified in the microarray experiment as more expressed. Click here for file

Additional file 3 **The fungal RcnAs form a distinct clade**. Phylogenetic analysis was carried out using the MEGA-2 (Molecular Evolutionary Genetics Analysis version 3.1) software (18, 2001; http://www.megasoftware.net). The SSR sequences were aligned and the dendrogram was determined by using the ClustalX and the Neighbor-Joining method, respectively (Saitou and Nei, 1987; Thompson et al., 1997). A bootstrap analysis (Felsenstein 1985) was performed (for 1,025 repeats) to evaluate the topology of the phylogenetic tree. The followings proteins were used forthe analysis: *Equus caballus *XP_001502684.1; *Macaca mulatta *XP_001102338.1; *Ornithorhynchus anatinu*s XP_001511608.1; *Gallus gallus *XP_420062.2; *Monodelphis domestica *XP_001363457.1; *Homo sapiens *NP_005813.2; *Bos taurus *NP_001015632.1; *Rattus norvegicus *NP_001012764.1; *Tetraodon nigroviridis *gi|47230037; *Xenopus tropicalis *gi|89272039|; *Xenopus laevis *NP_001080661.1; *Canis familiaris*. XP_858285.1; *Mus musculus *NP_062339.2; *Gorilla gorilla *gi|120975069|; *Macaca fascicularis *gi|90077144|; *Danio rerio *XP_001922378.1; *Apis mellifera *XP_396593.2; *Nasonia vitripennis *XP_001603743.1; *Tribolium castaneum *XP_969761.1; Drosophila mojavensis gi|193916784|; *Drosophila grimshawi *gi|193893692|; *Drosophila pseudoobscura *XP_001359704.1; *Drosophila erecta *gi|190651857|; Drosophila melanogaster NP_524378.1; *Brugia malayi *XP_001895925.1; *Malassezia globosa *XP_001730302.1; *Ustilago maydis *XP_756572.1; *Cryptococcus neoformans *XP_567126.1; *Laccaria bicolor *XP_001878504.1; *Coprinopsis cinerea *XP_001839847.1; *Yarrowia lipolytica *XP_503761.1; *Neosartorya fischeri *XP_001260765.1; *Aspergillus fumigatus Af293 *XP_755638.1; *Aspergillus clavatus *XP_001275581.1; *Aspergillus terreus *XP_001208640.1; *Aspergillus oryzae *XP_001821801.1; *Aspergillus niger *XP_001399317.1; *Aspergillus nidulans *XP_663853.1; *Coccidioides immitis *XP_001245666.1; *Ajellomyces capsulatus *XP_001541658.1; *Phaeosphaeria nodorum *XP_001797869.1; *Pyrenophora tritici-repentis *XP_001935909.1; *Botryotinia fuckeliana *XP_001554496.1; *Sclerotinia sclerotiorum *XP_001593917.1; *Chaetomium globosum *XP_001228347.1; *Neurospora crassa *XP_956953.1; *Magnaporthe grisea *XP_360675.1; *Gibberella zeae *XP_381626.1; *Podospora anserina *XP_001909744.1. References: Felsenstein J (1985); Confidence limits on phylogenies: an approach using the bootstrap. Evolution 39: 783-791. Saitou N, Nei M (1987); The neighbor-joining method: a new method for reconstructing phylogenetic trees. Mol Biol Evol 4: 406-425.
Thompson JD, Gibson TJ, Plewniak F, Jeanmougin F, Higgins DG (1997); The ClustalX windows interface: flexible strategies for multiple sequence alignment aided by quality analysis tools. Nucleic Acids Res. 24: 4876-4882. Click here for file

Additional file 4 **Primers used in this work**. List of primers used for PCRs and real time PCRs. Click here for file
