# Supplementary material for: Identification of possible targets of the Aspergillus fumigatus CRZ1 homologue, CrzA
Source: BMC Microbiol. 2010 Jan 15;10:12. doi: 10.1186/1471-2180-10-12 (PMC2818617; doi:10.1186/1471-2180-10-12)
Supplement: Additional file 1 — Genes less expressed after Aspergillus fumigatus ΔcrzA mutant CaCl2 200 mM exposition for 10 and 30 minutes. List of the genes identified in the microarray experiment as less expressed. [file 1471-2180-10-12-S1.PDF]

Table 1 – Genes less expressed after *Aspergillus fumigatus* Δ*crzA* mutant $CaCl_2$ 200 mM exposition for 10 and 30 minutes.

Gene	Log2 ratio	
	10’	30’
Inorganic ion transport and metabolism		
Afu2g05330 (KOG1397) vacuolar H+/Ca2+ exchanger	-1,22	ND
Afu3g10690 (KOG0204) calcium-translocating P-type ATPase(PMCA-type),putative	-0.71	-1.50
Afu7g01030 (KOG0204) Calcium-transporting ATPase 1 (PMC1), putative	-0.51	-1.00
General function prediction only		
Afu8g05010 (KOG1721) C2H2 finger domain protein, putative	ND	-1.30
Afu7g00120 (KOG0813) metallo-beta-lactamase domain protein, putative	-0.79	-1.27
Afu1g09700 (KOG0504) ankyrin repeat protein	ND	-1.00
Afu4g04320 (KOG0490) homeobox transcription factor	-0.73	-1.26
Cell wall/membrane/envelope biogenesis		
Afu2g01870 (KOG2571) chitin synthase A	-0.82	-1.23
Lipid transport and metabolism		
Afu7g00300 (KOG0497) Prenyltransferase and squalene oxidase repeat domain protein	-0.77	-1.67
Afu2g16520 (KOG1329) phospholipase D (PLD), putative	ND	-1.26
Intracellular trafficking, secretion, and vesicular transport		
Afu2g13760 (KOG3065) plasma membrane SNARE protein (Sec9), putative	-0.53	-1.07
Energy production and conversion		
Afu3g13440 (KOG2621) stomatin family protein	-0.61	-1.25
Cell cycle control, cell division, chromosome partitioning		
Afu5g08480 (KOG0590) serine/threonine protein kinase	-0.49	-1.14
Amino acid transport and metabolism		
Afu4g09840 (KOG3846) kynureninase	-0.66	-1.28
Function unknown		
Afu1g06850 (KOG2422) Nulp1-pending protein	-0.55	-1.00
Posttranslational modification, protein turnover, chaperones		
Afu5g13350 (KOG0883) peptidyl-prolyl cis-trans isomerase	ND	-1.27
Afu2g03720 (KOG0865) peptidyl-prolyl cis-trans isomerase	-0.60	-1.22
Signal transduction mechanisms		
Afu2g13060 (KOG4019) calcineurin binding protein	-0.58	-1.00
Nucleotide transport and metabolism		
Afu1g15830 (KOG1377) hypothetical protein	-0.84	-1.00

No KOG			
Afu6g10940	(No KOG) conserved hypothetical protein	-0.83	-1.71
Afu4g03170	(No KOG) hypothetical protein	-0.76	-1.31
Afu5g04290	(No KOG) WW domain protein, putative	-0.81	-1.27
Afu1g13230	(No KOG) conserved hypothetical protein	-0.75	-1.15
Afu3g13210	(No KOG) protein kinase domain-containing protein	-1.33	-1.72
Afu3g14230	(No KOG) BAR adaptor protein RVS167	-0.85	-1.00
Afu7g06040	(No KOG) integral membrane protein, putative	-0.39	-1.00
Afu3g09060	(No KOG) hypothetical protein	-0.46	-1.02
Afu4g03960	(No KOG) C6 transcription factor (Ctf1A), putative	-0.74	-1.01
Afu2g00580	(No KOG) hypothetical protein	-0.94	-1.00
Afu4g00980	(No KOG) hypothetical protein	-0.78	-1.04
Afu4g09330	(No KOG) conserved hypothetical protein	-0.57	-1.12
Afu4g10200	(No KOG) transcription factor RfeF, putative	ND	-1.20
