# Supplementary material for: Identification of possible targets of the Aspergillus fumigatus CRZ1 homologue, CrzA
Source: BMC Microbiol. 2010 Jan 15;10:12. doi: 10.1186/1471-2180-10-12 (PMC2818617; doi:10.1186/1471-2180-10-12)
Supplement: Additional file 2 — Genes more expressed after Aspergillus fumigatus ΔcrzA mutant CaCl2 200 mM exposition for 10 and 30 minutes. List of the genes identified in the microarray experiment as more expressed. [file 1471-2180-10-12-S2.PDF]

Table 2 – Genes more expressed after *Aspergillus fumigatus* *ΔcrzA* mutant $CaCl_2$ 200 mM exposition for 10 and 30 minutes.

Gene	Log2 ratio	
	10'	30'
RNA processing and modification		
Afu2g13980 (KOG0346) ATP dependent RNA helicase (Dbp9)	+1.08	ND
Afu6g11460 (KOG1295) related to nucleolar phosphoprotein	+0.47	+1.16
Afu2g05540 (KOG0050) cell division control protein (Cdc5)	+0.42	+1.00
Energy production and conversion		
Afu2g09130 (KOG3196) NADH-ubiquinone dehydrogenase 24 kDa subunit	+0.37	+1.00
Afu3g01460 (KOG2614) salicylate hydroxylase	+0.32	+1.00
Afu4g08240 (KOG1198) alcohol dehydrogenase, zinc-containing	+0.73	+1.72
Cell cycle control, cell division, chromosome partitioning		
Afu6g13160 (KOG0590) serine/threonine protein kinase	+0.80	+1.24
Afu3g14340 (KOG3285) mitotic spindle checkpoint protein (Mad2)	+0.92	+1.36
Afu5g08540 (KOG2655) septin	+1.50	+2.38
Amino acid transport and metabolism		
Afu1g17720 (KOG2551) oxidoreductase, putative	+0.74	+1.47
Afu3g07990 (KOG1289) GABA permease	+0.57	+1.81
Afu5g02180 (KOG1481) cysteine synthase (o-acetylserine (thiol)-lyase)	+0.50	+1.09
Afu4g12480 (KOG0573) asparagine synthase related protein	+0.66	+1.22
Afu3g11070 (KOG1184) pyruvate decarboxylase PdcA	+0.53	+1.27
Nucleotide transport and metabolism		
Afu7g06490 (KOG3981) deoxyribose-phosphate aldolase	+0.81	+1.58
Carbohydrate transport and metabolism		
Afu1g14560 (KOG2204) alpha-mannosidase	+0.81	+1.32
Afu4g02750 (KOG2504) related to monocarboxylate transporter	+0.52	+1.00
Afu7g08490 (KOG2806) class V chitinase	+0.84	+1.64
Lipid transport and metabolism		
Afu1g15170 (KOG3016) acyl-CoA thioesterase II	+0.65	+1.42
Afu1g06040 (KOG0380) sterol o-acyltransferase (APE2)	+0.41	+1.43
Afu4g11080 (KOG1175) acetyl-coenzyme A synthetase	+0.53	+1.18

Afu3g09240 (KOG3957) CAIB/BAIF family enzyme	+0.67	+1.06
Afu3g02570 (KOG1202) polyketide synthase	+0.46	+1.02
Translation, ribosomal structure and biogenesis		
Afu8g07110 (KOG0188) alanyl-tRNA synthetase	+0.44	+1.00
Afu5g04030 (KOG4600) mitochondrial ribosomal protein L2 precursor	+1.13	+2.16
Transcription		
Afu1g05830 (KOG0392) TBP associated factor (Mot1)	+0.88	+1.80
Afu2g16760 (KOG3152) conserved hypothetical protein	+0.76	+1.72
Afu2g07460 (KOG2712) RNA polymerase II transcriptional coactivator	+0.60	+1.00
Cell wall/membrane/envelope biogenesis		
Afu6g02460 (KOG3396) glucosamine 6-phosphate acetyltransferase	+0.77	+2.15
Posttranslational modification, protein turnover, chaperones		
Afu7g01230 (KOG0691) DnaJ domain protein	+0.59	+1.00
Afu4g04800 (KOG0730) AAA family ATPase, putative	+1.08	+1.45
Afu5g01750 (KOG1863) MATH and UCH domain protein	+0.40	+1.00
Inorganic ion transport and metabolism		
Afu6g00440 (KOG1485) cation diffusion facilitator 3	ND	+2.31
Secondary metabolites biosynthesis, transport and catabolism		
Afu2g09400 (KOG1399) cyclohexanone monooxygenase	ND	+1.00
Afu5g00310 (KOG1399) flavin-containing monooxygenase	+0.98	+1.06
Afu5g01360 (KOG0158) cytochrome P450	+1.23	+1.33
Afu8g06690 (KOG0157) cytochrome P450 alkane hydroxylase	+1.33	+2.34
General function prediction only		
Afu6g09970 (KOG0725) oxidoreductase, short chain dehydrogenase/reductase family	+0.85	+1.77
Afu7g01240 (KOG1382) phytase	+0.67	+1.00
Afu4g01140 (KOG0255) multidrug resistant protein	+0.27	+1.11
Afu1g08900 (KOG1940) CHY and RING finger domain protein	+0.72	+1.48
Afu6g12940 (KOG0813) metallo-beta-lactamase domain protein	+0.13	+1.27
Afu6g13030 (KOG0274) cell division control protein Cdc4	+0.65	+1.00
Afu1g02420 (KOG2950) conserved hypothetical protein	+1.24	+1.92
Afu3g13340 (KOG0393) related to transforming protein rho	+0.30	+1.00
Afu1g15010 (KOG3628) AMP binding domain protein	+0.32	+1.62
Function unknown		
Afu4g13270 (KOG3319) orm1 protein	+0.31	+1.00
Afu4g04470 (KOG4690) conserved hypothetical protein	+0.40	+1.12
Afu4g06560 (KOG2625) conserved hypothetical protein	+0.56	+1.01
Afu2g07540 (KOG3224) TOR signalling pathway protein TipA	+0.50	+1.00
Afu1g16610 (KOG4776) Swr1p complex component (Swc5)	+0.72	+1.11
Afu2g05010 (KOG0997) conserved hypothetical protein	+1.22	+2.51
Afu7g03960 (KOG4781) thioesterase family protein	+0.32	+1.14

Afu4g13180 (KOG3326) TPR repeat protein	+0.59	+1.00
Afu5g08660 (KOG2866) nuclear protein (Qri2)	+0.90	+2.08
Signal transduction mechanisms		
Afu4g11450 (KOG3519) Rho guanyl nucleotide exchange factor	+0.32	+1.02
Afu1g04700 (KOG3417) related to aimless RasGEF	+1,04	+1.86
Afu4g00720 (KOG0698) protein phosphatase 2C	+1.60	+2.59
Intracellular trafficking, secretion, and vesicular transport		
Afu5g02140 (KOG0566) inositol polyphosphate phosphatase	+0.88	+1.62
NO KOG		
Afu5g09600 (No KOG) 2-nitropropane dioxygenase family oxidoreductase	+0.52	+1.18
Afu5g09670 (No KOG) RNase III domain protein	+0.30	+1.07
Afu6g00620 (No KOG) GPI anchored hypothetical protein	+0.35	+1.44
Afu6g12810 (No KOG) hypothetical protein	+0.52	+1.40
Afu7g05060 (No KOG) cation transport ATPase yqgG	+0.66	+1.24
Afu8g00230 (No KOG) phytanoyl-CoA dioxygenase family protein	+0.68	+1.16
Afu6g13150 (No KOG) hypothetical protein	+1.27	+2.48
Afu7g01200 (No KOG) aspergillopepsin	+0.95	+1.57
Afu8g01370 (No KOG) methyltransferase	+0.58	+1.34
Afu8g07310 (No KOG) hypothetical protein	+0.49	+1.27
Afu8g00820 (No KOG) rhamnogalacturonase	+1.42	+2.82
Afu8g01190 (No KOG) MitR	+0.98	+1.54
Afu6g03450 (No KOG) conserved hypothetical protein	+0.20	+1.06
Afu6g07410 (No KOG) hypothetical protein	+1.37	+2.24
Afu6g09353 (No KOG) hypothetical protein	+1.02	+1.80
Afu6g04070 (No KOG) hypothetical protein	+1.10	+2.18
Afu6g06700 (No KOG) hypothetical protein	+0.79	+1.17
Afu6g11860 (No KOG) hypothetical protein	+1.06	+2.11
Afu6g12180 (No KOG) conserved hypothetical protein	ND	+1.16
Afu6g11760 (No KOG) hypothetical protein	+0.90	+2.02
Afu6g11820 (No KOG) Glycosyl hydrolase family 10	+0.88	+1.75
Afu1g13830 (No KOG) hypothetical protein	+1.06	+1.59
Afu1g17580 (No KOG) xenobiotic compound monooxygenase, DszA family	+0.83	+1.60
Afu2g00430 (No KOG) exo-beta-1,3-glucanase	+0.75	+1.00
Afu1g16090 (No KOG) arsenate reductase (ArsC)	+0.91	+1.65
Afu1g17370 (No KOG) scf1 protein	+0.66	+1.80
Afu2g05410 (No KOG) hypothetical protein	+0.83	+1.67
Afu3g05870 (No KOG) IFRD domain protein	+0.82	+1.43
Afu2g01340 (No KOG) hypothetical protein	+0.61	+1.12
Afu2g03830 (No KOG) allergen Asp F4	+0.62	+1.33
Afu1g00110 (No KOG) hypothetical protein	+0.33	+1.10
Afu1g04480 (No KOG) Sin3 complex subunit (Stb2)	+1.32	+1.77

Afu1g05210 (No KOG) G-protein complex gamma subunit	+0.59	+1.03
Afu1g00190 (No KOG) hypothetical protein	+0.28	+1.95
Afu1g01410 (No KOG) hypothetical protein	ND	+1.70
Afu1g11740 (No KOG) hypothetical protein	+0.55	+1.01
Afu1g12420 (No KOG) hypothetical protein	+0.51	+1.25
Afu1g07430 (No KOG) hypothetical protein	+0.43	+1.30
Afu1g09190 (No KOG) C6 finger domain protein	+0.62	+1.12
Afu4g03480 (No KOG) hypothetical protein	+0.50	+1.57
Afu4g06510 (No KOG) conserved hypothetical protein	+0.49	+1.01
Afu3g05910 (No KOG) hypothetical protein	+0.69	+1.76
Afu3g09260 (No KOG) hypothetical protein	+1.03	+1.95
Afu5g01140 (No KOG) plasma membrane ammonium transporter (Ato3)	+0.35	+2.31
Afu5g09380 (No KOG) hypothetical protein	+0.57	+1.24
Afu4g09550 (No KOG) hypothetical protein	+0.50	+1.09
Afu5g00820 (No KOG) hypothetical protein	+1.61	+2.85
Afu2g07390 (No KOG) hypothetical protein	+0.41	+1.02
Afu2g14870 (No KOG) peptidase, M23/M37 family	+0.70	+2.04
Afu2g17900 (No KOG) conserved hypothetical protein	+0.49	+1.15
