# Supplementary figures and images for: Identification of possible targets of the Aspergillus fumigatus CRZ1 homologue, CrzA
Source: BMC Microbiol. 2010 Jan 15;10:12. doi: 10.1186/1471-2180-10-12 (PMC2818617; doi:10.1186/1471-2180-10-12)

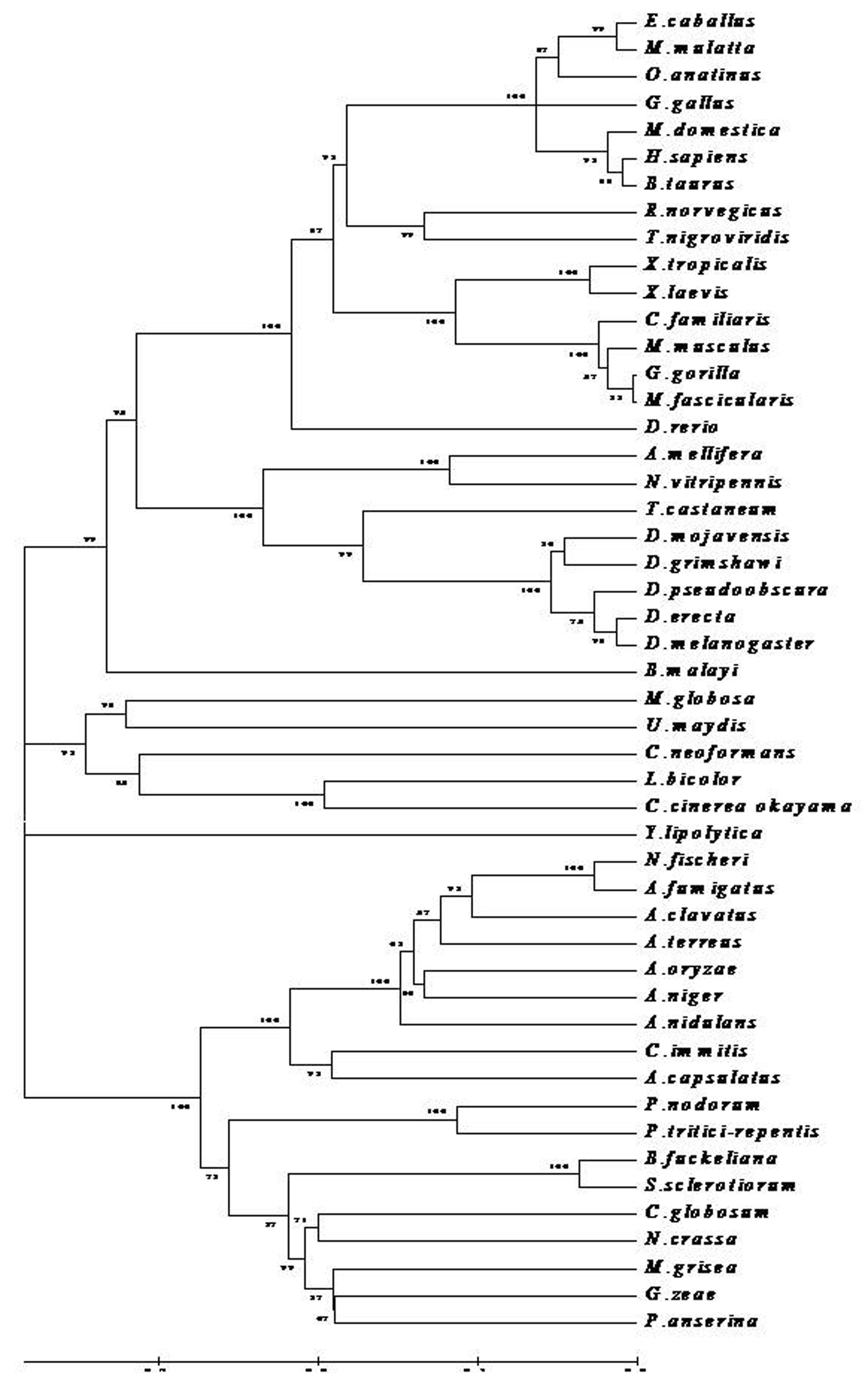

Supplement: Additional file 3 — The fungal RcnAs form a distinct clade. Phylogenetic analysis was carried out using the MEGA-2 (Molecular Evolutionary Genetics Analysis version 3.1) software (18, 2001; http://www.megasoftware.net). The SSR sequences were aligned and the dendrogram was determined by using the ClustalX and the Neighbor-Joining method, respectively (Saitou and Nei, 1987; Thompson et al., 1997). A bootstrap analysis (Felsenstein 1985) was performed (for 1,025 repeats) to evaluate the topology of the phylogenetic tree. The followings proteins were used forthe analysis: Equus caballus XP_001502684.1; Macaca mulatta XP_001102338.1; Ornithorhynchus anatinus XP_001511608.1; Gallus gallus XP_420062.2; Monodelphis domestica XP_001363457.1; Homo sapiens NP_005813.2; Bos taurus NP_001015632.1; Rattus norvegicus NP_001012764.1; Tetraodon nigroviridis gi|47230037; Xenopus tropicalis gi|89272039|; Xenopus laevis NP_001080661.1; Canis familiaris. XP_858285.1; Mus musculus NP_062339.2; Gorilla gorilla gi|120975069|; Macaca fascicularis gi|90077144|; Danio rerio XP_001922378.1; Apis mellifera XP_396593.2; Nasonia vitripennis XP_001603743.1; Tribolium castaneum XP_969761.1; Drosophila mojavensis gi|193916784|; Drosophila grimshawi gi|193893692|; Drosophila pseudoobscura XP_001359704.1; Drosophila erecta gi|190651857|; Drosophila melanogaster NP_524378.1; Brugia malayi XP_001895925.1; Malassezia globosa XP_001730302.1; Ustilago maydis XP_756572.1; Cryptococcus neoformans XP_567126.1; Laccaria bicolor XP_001878504.1; Coprinopsis cinerea XP_001839847.1; Yarrowia lipolytica XP_503761.1; Neosartorya fischeri XP_001260765.1; Aspergillus fumigatus Af293 XP_755638.1; Aspergillus clavatus XP_001275581.1; Aspergillus terreus XP_001208640.1; Aspergillus oryzae XP_001821801.1; Aspergillus niger XP_001399317.1; Aspergillus nidulans XP_663853.1; Coccidioides immitis XP_001245666.1; Ajellomyces capsulatus XP_001541658.1; Phaeosphaeria nodorum XP_001797869.1; Pyrenophora tritici-repentis XP_001935909.1; Botryotinia [file 1471-2180-10-12-S3.JPEG]
