# Supplementary material for: Identification of possible targets of the Aspergillus fumigatus CRZ1 homologue, CrzA
Source: BMC Microbiol. 2010 Jan 15;10:12. doi: 10.1186/1471-2180-10-12 (PMC2818617; doi:10.1186/1471-2180-10-12)
Supplement: Additional file 4 — Primers used in this work. List of primers used for PCRs and real time PCRs. [file 1471-2180-10-12-S4.PDF]

Table 3 - Primers used in this work.

Name	Sequence
pyrG Fw	5'- CTTGCAGATACTAAAGAATT -3'
pyrG Rw	5'- ACCACAGACGAAGACGGAAG -3'
calp-Ani P1	5'- GTAACGCCAGGGTTTTCCCAGTCACGACGCGTTCCGTCAAATCATTGAC -3'
calp-Ani P2	5'- GTGCCTCCTCTCAGACAGAATAATTCTTTAGTATCTGCAAG –3'
calp-Ani P3	5'- AGCATTGTTTGAGGCGAATTCGCTTTTGACGTCTTGAGTAC –3'
calp-Ani P4	5'-GCGGATAACAATTTCACACAGGAAACAGCCGTACTCGTATTTTAAATCC-3'
calp-Afu P1	5'-GTAACGCCAGGGTTTTCCCAGTCACGACGAACTTCTCTACTTATCAAGC-3'
calp-Afu P2	5'-GTGCCTCCTCTCAGACAGAATGCAATTGGAAGTAATTGATGC –3'
calp-Afu P3	5'- AGCATTGTTTGAGGCGAATTCTCATCGATGGTCTTTTTCCC –3'
calp-Afu P4	5'-GCGGATAACAATTTCACACAGGAAACAGCGGAACAAATTCGTTCTGGGTC-3'
AN calcip AscI	5'-GGCGCGCCAATGCATCAACGCAACGAAAT-3'
AN calcip PacI	5'-CCTTAATTAATCAAACCATCAACTCAACAGG-3'
PMC1a_3614RL	CGGACCCTGGAAACGAACTCGTC[FAM]G*
PMC1a_3614RL/3591FU	GGCGTGATTTCTCTGCCTGTC
PMC1b_3488RL	CGGAACTGTCGTGGAGTCGTCTTC[FAM]G
PMC1b_3488RL/3454FU	TCGTCTATGATGTGGCCTGTCA
Fosfolipase D Afu2g16520_2494RL	CGGTGCTCGCTAACAGTATCCCAC[FAM]G
Fosfolipase D Afu2g16520_2494RL/2472FU	CAGGCTGCTCATCAAGTGTCG
calA binding prot Afu2g13060_175FL	CGGTTCCTCAAACACGCTACTAATAAC[FAM]G
calA binding prot Afu2g13060_175FL/190RU	GGGAGGCCGGTTGAAAGAGGTA
C6 finger domain Afu2g11460_291RL	CGGCTATACGCTCCGAAGATGC[FAM]G
C6 Finger domain Afu2g11460_291RL/273FU	GCAAGGGCTATATCCAGCGTGTC
β-tubulin 634FL	CGAGCCCTCTCGTTCACCAGCT[FAM]G'
β-tubulin 663RU	GTCGTACAGAGCCTCGTTGTCG
Afu3g14230 BAR_1109RL	CGGGTATAATCTCAATCACGTCACC[FAM]G
Afu3g14230 BAR_1109RL/1060FU	CGGCAGTCGAGACGGTTACAG
Afu crzA_849RL	CGCTGGGTACGGAGAAGGAGCAG[FAM]G
Afu crzA_849RL/795FU	AGGCCGTCATGGGCAATAGT
Afu4g10200 RFeF_458RL	CGGCGAGAGTGGGATGTAGGTGC[FAM]G
Afu4g10200 RFeF_458RL/406FU	CCGCAACAGCAACAATATCAGC
AAA ATPase Afu4g04800_100FL	CGGCCTCATCATCGTCCCTAGC[FAM]G

AAA ATPase Afu4g04800_100FL/121RU	GCGTGTTCACACTGCAAATGTC
scf1 Afu1g17370_97FL	CGGTTATGGACAAGATGAAGGAAAC[FAM]G
scf1 Afu1g17370_97FL/147RU	GCCTGCTGCGAGTACGACTTT
AN calcipressin_429RL	CGGCGGAGCTTATGCTTGATGC[FAM]G
AN calcipressin_429RL/391FU	CCGCACACGGATCTACTTCG

* FAM: 6-carboxyfluorescein
